# Detection Rate and Clinical Relevance of Ink Tattooing during Balloon-Assisted Enteroscopy

**DOI:** 10.1155/2017/4969814

**Published:** 2017-11-05

**Authors:** C. Römmele, A. Ebigbo, M. Schrempf, H. Messmann, S. K. Gölder

**Affiliations:** ^1^Department of Internal Medicine III, Klinikum Augsburg, Augsburg, Germany; ^2^Department of General, Visceral and Transplantation Surgery, Klinikum Augsburg, Germany

## Abstract

**Background and Aims:**

Balloon-assisted enteroscopy (BAE) is a well-established tool in the diagnosis and therapy of small bowel diseases. Ink tattooing of the small bowel is used to mark pathologic lesions or the depth of small bowel insertion. The purpose of this study was to determine the safety, the detection rate, and the clinical relevance of ink tattooing during BAE.

**Methods:**

We performed a retrospective analysis of all 81 patients who received an ink tattooing during BAE between 2010 and 2015.

**Results:**

In all patients, ink tattooing was performed with no complications. 26 patients received a capsule endoscopy after BAE. The tattoo could be detected via capsule endoscopy in 19 of these 26 patients. The tattoo of the previous BAE could be detected via opposite BAE in 2 of 11 patients. In 9 patients, ink tattooing influenced the choice of approach for reenteroscopy. In 7 patients, the tattoo was used for intraoperative localization and in 3 patients for intraoperative localization as well as for reenteroscopy. The intraoperative detection rate of the tattoo was 100%.

**Conclusion:**

Ink tattooing of the small intestine is a safe endoscopic procedure to mark the depth of scope insertion or a pathologic lesion during balloon-assisted enteroscopy.

## 1. Introduction

Endoscopic tattooing is an old and well-established tool for marking of pathologic lesions in the colon. The technique was first described for sigmoidoscopy by Sauntry and Knudston in 1958 [1]. Tattooing with India ink was first described in 1975 [2]. Over the years, the technique was improved and has been shown to be a safe procedure. Complications occur only rarely [3]. The main complications described in the literature are associated with the transmural injection of the ink inducing inflammatory processes such as peritonitis, small bowel infarction, and abscesses [2, 4].

Double-balloon-assisted enteroscopy (DBE) was developed in 2001 by Yamamoto. It makes a complete enteroscopy with therapeutic intervention in the small intestine possible [5, 6]. Due to missing landmarks of the small intestine, India ink was used for small bowel tattooing. By using ink tattooing as a landmark, Yamamoto et al. were able to demonstrate complete enteroscopy by an antegrade and a retrograde approach in 24 of 28 patients (86%) [5]. Single-balloon enteroscopy (SBE) was introduced in 2007 with similar insertion depths and adverse events as compared to DBE [7, 8]. The main indications for performing an enteroscopy of the small intestine are suspected or known gastrointestinal bleeding and for the evaluation of suspected or known polyps or tumors of the small intestine. Other indications include the DBE-assisted endoscopic retrograde cholangiopancreatography (ERCP) in patients with altered upper gastrointestinal anatomy, such as Roux-en-Y gastric bypass [9].

However, adequate data concerning ink tattooing of the small intestine has not yet been published.

The purpose of this retrospective study was to examine the safety, the detection rate during a surgical operation or video capsule endoscopy, and the clinical relevance of ink tattooing during balloon-assisted enteroscopy (BAE).

## 2. Methods

### 2.1. Study Population

All patients who received a BAE from January 01, 2010, to December 31, 2015, were considered eligible for inclusion in this study. The inclusion criterion was an ink tattooing of the small intestine during the examination. The study was conducted in accordance with the ethical principles of the Declaration of Helsinki and in compliance with good clinical practice and local regulations.

### 2.2. Endoscopic Procedure

An informed consent was obtained from all patients after providing them with adequate information on possible complications associated with the procedure such as dental injuries, bleeding, perforation, cardiovascular failure due to sedation, and allergic reaction. All patients were sedated with midazolam and when necessary, in combination with Disoprivan (propofol). In some cases, additional pethidine was given.

### 2.3. Ink Tattooing Procedure

**Figure 1:**
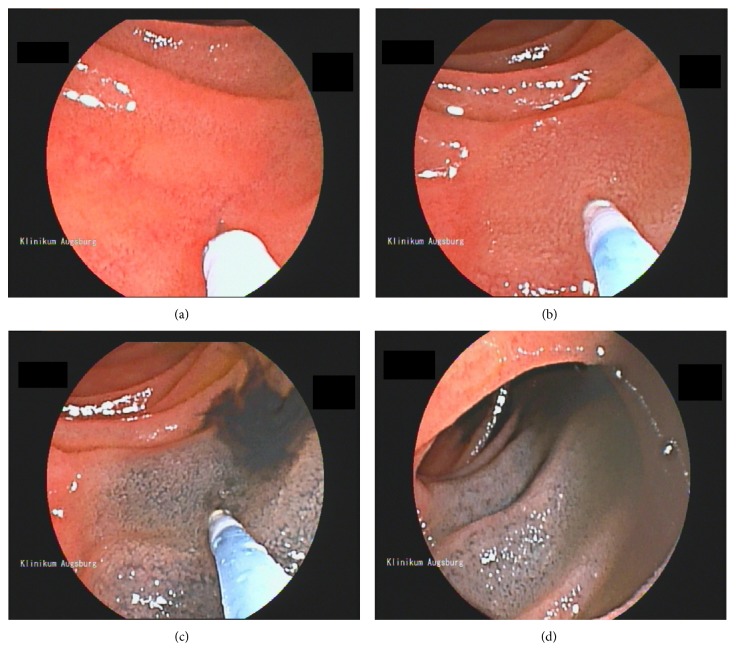
Technique of ink injection. Ink tattooing of the small bowel during a balloon-assisted enteroscopy. The injection needle is placed on the small bowel surface (a). After penetration of the mucosal layer of the small intestine, saline solution is injected and a submucosal lifting can be observed (b). Subsequently, the ink is injected into the submucosal cushion and under endoscopic view (c). After ink injection, the needle is retracted and the tattoo is documented (d).

Ink tattooing was performed with an injection needle through the working channel of the endoscope. The InjectorForce Max (Olympus, Hamburg Germany) with a working length of 2700 mm, needle diameter of 0.6 mm, and a needle length of 4 mm as well as the MANTA injection needle (Medwork, Höchstadt, Germany) with a working length of 2400 mm, needle diameter 0.7 mm, and a needle length of 5 mm was used. First, the needle was flushed with saline solution. Then, the needle was advanced to the submucosal layer of the small bowel. The first injection was performed with saline solution until a submucosal cushion was observed. The ink was then injected into the submucosal cushion thereby reducing the risk of a transmural administration of the ink. With this method, inflammatory complications such as abscess or peritonitis become unlikely. A few milliliters of injected ink was usually sufficient for the tattooing procedure (Figure 1). If a tumor or polyp was marked with an ink tattoo, the tattoo was regularly placed proximal to the lesion site.

We used the ink Black Eye (The Standard, Seoul, Korea), which, in contrast to original nonsterile India ink, is sterile. Furthermore, it has a high purity of suspended carbon particles, whereas India ink often contains impurities such as phenols, ammonia, and animal products.

The material costs for the application of an ink tattoo in one patient, including a needle, ink, and saline solution, amount to about 50€ (60 US$) without tax.

### 2.4. Analysis

A clinical or therapeutic consequence of the ink tattooing procedure was assumed when the tattoo was used during surgery to identify the pathologic lesion which had been marked previously. Also, when the ink tattoo influenced the choice of a subsequent endoscopic examination as was the case in patients with a second bleeding episode in whom a capsule endoscopy and then a second BAE was performed within the study period (January 01, 2010, to December 31, 2015).

## 3. Results

The present study is a retrospective open-label single-center analysis at Klinikum Augsburg. Between January 01, 2010, and December 31, 2015, 229 BAE were performed in 156 patients at the endoscopy unit of Klinikum Augsburg. 26 of these 229 examinations were performed as a single-balloon enteroscopy, the rest was done using the double-balloon technique. 171 BAE were performed with an antegrade and 58 with a retrograde approach. The indications for BAE were Crohn's disease (20 patients), angiodysplasia (47 patients), suspected gastrointestinal bleeding (43 patients), anemia (30 patients), known or suspected tumor or polyps (30 patients), and other indications such as Meckel's diverticulum, invagination, unspecified ulcers, foreign bodies, stenosis of the small intestine, and search of the major duodenal papilla after a gastrojejunostomy (30 patients).

**Figure 2:**
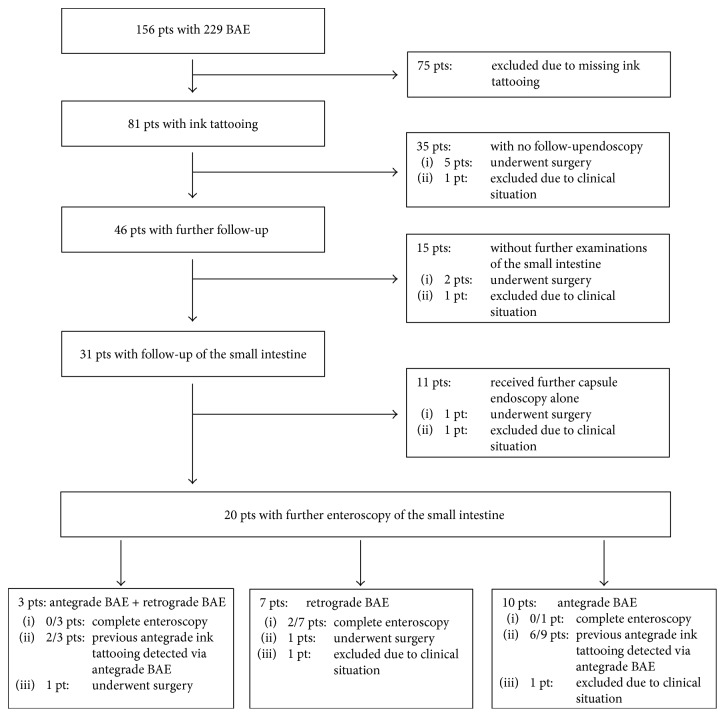
Flowchart of 229 balloon-assisted enteroscopies in 156 patients for small intestine diagnostics at our endoscopic unit. BAE: balloon-assisted enteroscopy; pts: patients.

11 patients had to be excluded because the BAE achieved no deeper intubation of the small intestine with its push-and-pull principle compared to the conventional endoscopy. Four patients had to be excluded because of a respiratory depression under sedation. One patient was excluded because of age under 18 years. In 59 BAE, no ink tattooing was performed and the patients were therefore excluded. Thus, 81 patients met the entry criteria. 73 patients received a BAE with an antegrade and eight patients with a retrograde approach. The median age of the patients was 69 years (range 23–92). In all 81 patients, ink tattooing of the small intestine was performed without any complications. Five patients had to be excluded over the course of time (see also Figure 2). Two patients aborted their stay in our hospital, and three patients suffered under severe worsening of their health condition so that in accordance with their will no further diagnostic or therapeutic steps were taken.

In 27 patients (33%), no pathologic finding was discovered during the BAE examination. In 35 patients, angiodysplasia (43%) was found and treated in 26 cases with APC and in nine cases with a clip. Further findings were hemangioma (2 patients, 2%), ulcers and other inflammatory lesions (7 patients, 9%), and tumor and polyps (11 patients, 14%) as well as ulcerative diverticula (1 patient, 1%). Two patients showed two different pathologic findings during the BAE; in four patients, the pathologic lesions showed an active bleeding (see Table 1).

Follow-up was defined as any further endoscopic examination of the gastrointestinal tract (including capsule enteroscopy) within the study period. No follow-up was performed in five patients who underwent surgery directly after enteroscopy and in one patient due to his poor clinical condition.

46 of 81 patients (57%) received further examinations. Further diagnostic was needed in 24 patients due to a rebleeding episode and in 6 patients for follow-up monitoring of a polyp/tumor. In 11 patients, a further examination was indicated as a second look for completion of the APC therapy or for an approach from the opposite side. One patient needed a second examination because of Crohn's disease and in four cases, the suspected pathologic lesion was not found in the BAE (see Figure 2).

**Figure 3:**
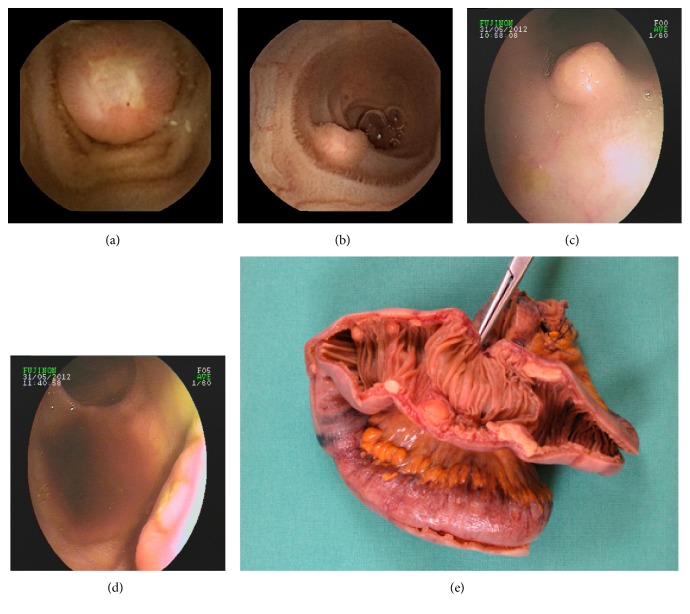
Typical case report. A 64-year-old woman was transferred to our hospital with suspected midgastrointestinal bleeding. The patient had a history of a MALT lymphoma. An externally performed colonoscopy as well as gastroscopy showed no pathologic finding. A capsule endoscopy was performed (a and b) showing a submucosal tumor with an ulceration on the surface. A DBE with an antegrade approach showed no pathologic findings. In the retrograde DBE, several submucosal tumors could be found (c and d). The histological examinations showed a carcinoid tumor. The patient received a resection of a small bowel segment after a DOTATE-PET-CT scan was performed (e). (a and b) Pictures of the findings of the video capsule endoscopy balloon-assisted enteroscopy. (c and d) Pictures of the retrograde DBE. (e) Intraoperative preparation. Clearly visible is the ink tattoo on the left side which was used as an intraoperative landmark.

15 of 46 received no further examination of the small intestine. Two patients received no further diagnostic examination of the small intestine because they underwent surgery and one because of his poor clinical condition (see Figure 2). A typical case report is shown in Figure 3.

31 patients received further examinations of the small intestine, and 20 patients with a second BAE. 13 patients were followed up with a BAE using an antegrade approach. 12 of these patients had already received an antegrade BAE. One of these patients initially received a retrograde BAE. The ink tattoo of the previous BAE was reached in eight cases with the following BAE; in three cases, a further ink tattooing was performed. One patient received an antegrade BAE after an initial retrograde approach. The ink tattoo was not reached and a new tattoo was placed. 15 patients were planned for a BAE with a retrograde approach as a follow-up examination. However, in five patients, intubation of the terminal ileum during retrograde BAE was not achieved. The remaining ten patients had initially had a BAE with an antegrade approach. The ink tattoo of the previous opposite BAE was reached in only two patients. Five patients received a second ink tattoo. Three patients underwent surgery after these further diagnostic steps, and another three patients had to be excluded due to their clinical situation (see Figure 2).

**Figure 4:**
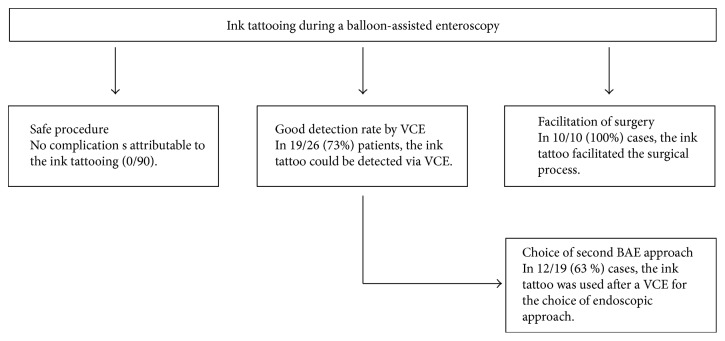
Flowchart of the main findings of the study. BAE: balloon-assisted enteroscopy; pts: patients, VCE: video capsule endoscopy.

In total, 26 patients received a capsule endoscopy after BAE with ink tattooing. In 19 of these 26 patients (73%), the ink tattoo was detected in the capsule endoscopy. The detection of the tattoo via VCE determined the approach of reenteroscopy in 12 of 19 cases (63%) (see Figure 4).

In summary, 90 ink tattooing procedures were performed in 81 patients without any complications. Ink tattooing had a clinical or a therapeutic consequence in 19 of 81 patients (23%). In 12 patients, the ink tattoo was used in the follow-up for the choice of an antegrade or retrograde approach. In ten cases, the ink tattoo facilitated the localization of the area of interest during surgery (see Figure 4). Nine of these ten patients who underwent surgery had a tumor of the small intestine, and active bleeding was seen in two patients. One patient was operated due to recurrent bleeding from hemangioma in the small intestine. In three patients, the ink tattoo was used for further endoscopies as well as for surgery over the course of time. The ink tattoo could be detected via capsule endoscopy in 73% of cases (see Figure 4). A complete enteroscopy was achieved only in two of 11 patients (18%) with an antegrade as well as a retrograde approach. Neither patients had a history of abdominal surgery.

## 4. Discussion

India ink for tattooing of the gastrointestinal tract was first used in the colon, where it was shown to be a safe procedure with a clinical complication rate below 1% [1, 3]. At the introduction of the DBE by Yamamoto et al., India ink was used for marking the depth of insertion into the small intestine [5]. Since then, ink tattooing of the small intestine has been used routinely for marking pathologic lesions or the depth of insertion. However, no adequate data concerning ink tattooing of the small intestine has been published so far. Ink tattooing was performed in our study 90 times in the small intestine without any complications. Our study is the first study showing the safety of ink tattooing of the small intestine.

A large meta-analysis in 2016 showed that ink tattooing of colorectal cancers within a colonoscopy leads to fewer localization errors during surgery. Therefore, the authors recommend a routine use of ink tattooing of pathologic lesions in the colon prior to operation [10]. In our study, the intraoperative localization of the pathologic lesion was facilitated by the earlier performed ink tattoo. In all ten cases (100%) that underwent surgery, the area of interest was recognized by the surgeon due to the ink tattoo. Similar to the recommendation of Acuna et al. [10] in the colon, we suggest ink tattooing of all tumors of the small intestine detected during enteroscopy.

The most common indication for an examination of the small intestine is gastrointestinal bleeding with 60–70% of cases [11, 12]. This rate is in accordance to our findings with 77% gastrointestinal bleeding, anemia, or known angiodysplasia. Our data shows that an ink tattoo is especially helpful in such patients with angiodysplasia or bleeding hemangioma. If rebleeding occurs, the marking can be used after a video capsule endoscopy for the choice of approach for subsequent balloon-assisted enteroscopy. This was the case in the majority (63%) of ink tattoos detected via VCE in our study.

The rate of complete visualization of the small intestine with balloon enteroscopy with an antegrade and retrograde approach was 18% in our study. This rate differs largely form initially published data, where a complete visualization rate of 86% for DBE was achieved [5]. However, results of further published data show completion rates comparable to our results [13–16]. Our retrospective data indicates that in clinical reality and beyond the setting of study situations such a high completion rate seem unlikely. Most of the available literature indicates a better complete visualization rate of the small intestine for DBE compared to SBE [6, 7, 16–20]. However, it remains a subject of ongoing discussions, whether a complete enteroscopy is necessary to improve the diagnostic yield or to influence further therapeutic decisions [18, 20–22]. Nonetheless, a better and more complete inspection with the possibility of immediate endoscopic treatment of findings could be relevant for the outcome of patients suffering from such ailments as angiodysplasia or polyposis syndromes [21].

Adverse events through a DBE (1.6%) or SBE (2.2%) examination occur only rarely [7]. In our study, no adverse events occurred as a result of enteroscopy.

Beside the balloon-assisted push-and-pull-principle, another principle used is the so-called spiral enteroscopy (SE). The principal of SE involves scope insertion by rotation of a flexible overtube which is placed over a standard enteroscope. Initial data has shown lower or equal complete visualization rates for the SE compared to the DBE [16, 23]. A new endoscope with an integrated motorized spiral system has been developed for spiral enteroscopy and is in the phase of clinical testing [11]. It is unclear how significant the impact of SE will be on the completion rate in clinical reality.

The study limitations are its retrospective, electronic data-based, and single-center design. Therefore, ink tattooing of the small intestine may actually have a higher rate of clinical or therapeutic consequences than the 23% shown in this study. The reason for this assumption is based on the fact that due to the study design, the follow-up period may have been too short and some patients may not have been observed sufficiently.

## 5. Conclusion

In summary, ink tattooing of the small intestine is a minimally invasive and safe endoscopic procedure to mark the depth of scope insertion or a pathologic lesion. It is a useful tool which facilitates the intraoperative localization of pathologic lesions and influences the choice of approach of a reenteroscopy, when indicated. The detection rate of the ink tattoo with the video capsule was significant. The intraoperative detection rate of the ink tattoo was 100%. A complete enteroscopy of the small intestine via BAE from a retrograde and an antegrade approach was rarely achieved in our clinical setting.

## Figures and Tables

**Table 1:** Demographic and clinical characteristics.

Parameter	
Age (years)	69 [23–92]
Sex (male/female)	58/23
BAE with primary ink tattooing(antegrade/retrograde)	81 [73/8]
No pathologic finding	27 (33%)
Angiodysplasia	35 (43%)
Hemangioma	2 (2%)
Ulcers/inflammatory lesions	7 (9%)
Tumor/polyps	11 (14%)
Ulcerative diverticula	1 (1%)
Two pathologic findings within the BAE	2
Active bleeding of the pathologic finding	4
